# Two-phase learning-based 3D deblurring method for digital breast tomosynthesis images

**DOI:** 10.1371/journal.pone.0262736

**Published:** 2022-01-24

**Authors:** Yunsu Choi, Minah Han, Hanjoo Jang, Hyunjung Shim, Jongduk Baek

**Affiliations:** School of Integrated Technology and Yonsei Institute of Convergence Technology, Yonsei University, Incheon, South Korea; Fuzhou University, CHINA

## Abstract

In digital breast tomosynthesis (DBT) systems, projection data are acquired from a limited number of angles. Consequently, the reconstructed images contain severe blurring artifacts that might heavily degrade the DBT image quality and cause difficulties in detecting lesions. In this study, we propose a two-phase learning approach for artifact compensation in a coarse-to-fine manner to mitigate blurring artifacts effectively along all viewing directions of the DBT image volume (i.e., along the axial, coronal, and sagittal planes) to improve the detection performance of lesions. The proposed method employs a convolutional neural network model comprising two submodels/phases, with Phase 1 performing three-dimensional (3D) deblurring and Phase 2 performing additional 2D deblurring. To investigate the effects of loss functions on the proposed model’s deblurring performance, we evaluated several loss functions, such as the pixel-based loss function, adversarial-based loss function, and perception-based loss function. Compared with the DBT image, the mean squared error of the image and the root mean squared errors of the gradient of the image decreased by 82.8% and 44.9%, respectively, and the contrast-to-noise ratio increased by 183.4% in the in-focus plane. We verified that the proposed method sequentially restored the missing frequency components as the DBT images were processed through the Phase 1 and Phase 2 steps. These results indicate that the proposed method performs effective 3D deblurring, significantly reducing the blurring artifacts in the in-focus plane and other planes of the DBT image, thus improving the detection performance of lesions.

## Introduction

Digital breast tomosynthesis (DBT) imaging systems widely used for chest, wrist, head, neck, dental and breast for medical diagnostics [1–5]. Recent developments in high-quality digital receptors have allowed DBT systems to be used in detecting breast cancer [5, 6]. Unlike mammograms, DBT systems use multiple projection data from different viewing angles, resulting in a significant improvement in detection accuracies in reconstructed DBT images [7, 8].

**Fig 1:**
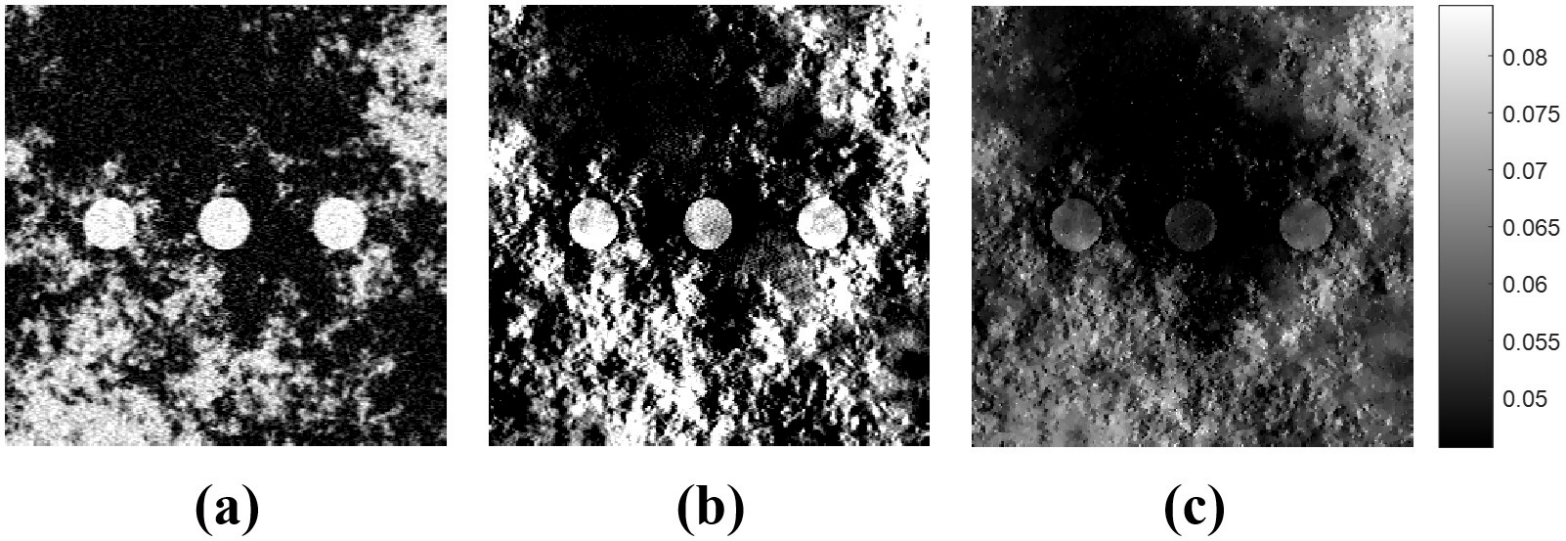
Limitation of the conventional DBT reconstruction algorithm. (a) CBCT images, (b) DBT images reconstructed using FDK, and (c) DBT images reconstructed using GP-BB. The display window is [0.0456 0.0844] *cm*^−1^.

As the DBT system obtains patient data scanned from a limited range of angles (e.g., 30° to 60° [9]), severe blurring artifacts occur when conventional filtered backprojection methods are used for image reconstruction (e.g., the Feldkamp–Davis–Kress (FDK) [10] algorithm). Although analysis-based methods, such as the gradient-projection Barzilai-Borwein algorithm (GP-BB) [11], have been developed to improve the image quality of DBT systems, they still have limitations in terms of reducing the blurring artifacts, especially in breast tissue images, as illustrated in Fig 1.

In recent studies, convolutional neural networks (CNNs) for mitigating blurring artifacts caused by camera motion [12] have been proposed. The camera motion is formulated as convolutions between the reference images and motion kernels. Theoretically, considering that the FDK algorithm is a linear system, the DBT image reconstructed by FDK algorithm can be expressed as a convolution between the reference image and the point spread function (PSF) [13], similar to camera motion deblurring as follows:

$$\begin{array}{c} \begin{array}{c} r(x,y,z)=i(x,y,z)***p(x,y,z)+n(x,y,z) \end{array} \end{array}$$ (1)

where *i*(*x*, *y*, *z*) is an ideal breast image, *p*(*x*, *y*, *z*) is the 3D PSF of DBT system, *r*(*x*, *y*, *z*) is the reconstructed DBT image, and *n*(*x*, *y*, *z*) is the reconstructed noise. The conventional deconvolution method such as Richardson-Lucy (RL) [14], which requires a manual control of the parameters in RL deconvolution, is not suitable for deblurring DBT image because accurate estimation of the PSF is difficult [13]. However, due to the robust characteristics of CNN and its wide receptive field, a more accurate deblurring kernel with spatially varying properties can be estimated. In our previous work [15], we proposed a method to deblur DBT images using a deep residual-block-based CNN (DRCNN), where the cone-beam computed tomography (CBCT) images reconstructed by the FDK algorithm were used as target images. As the CBCT data were acquired over a 360° range, the reconstructed image did not contain blurring artifacts caused by insufficient view sampling. Our previously proposed CNN learned the local and global properties of the blurring artifacts; thus, it reduced these blurring artifacts effectively in the in-focus plane using the two-dimensional (2D) in-focus slice data for training the model. However, the blurring artifacts could not be effectively reduced in the images along the coronal and sagittal planes because the DBT system captures less sufficient data along the coronal and sagittal planes. To solve the afore-mentioned problem, a new method for 3D deblurring the DBT volume is required.

In this study, we propose a two-phase learning-based 3D deblurring technique to reduce the blurring artifacts along all imaging planes of DBT images from anatomical backgrounds. As the DBT system produces much blurrier images along the coronal plane, we designed our network to reflect this spatially varying property of DBT images for effective deblurring. Our proposed two-phase learning method involves two different network models with a sequential training scheme. In Phase 1, we perform an initial 3D deblurring on the 3D DBT volume, where the entire volume is restored at a coarse scale. In Phase 2, we increase the sharpness of the restored 3D volume obtained from Phase 1 by applying U-Net [16] along the coronal plane, where the blurring artifacts are observed to be most severe.

To investigate the effects of loss functions on our model’s deblurring performance, we evaluate various loss functions (i.e., pixel-based loss, adversarial-based loss [17], and perception-based loss [18]). Pixel-based evaluations are conducted using the mean squared error of the image (MSE) and the root mean squared errors of the gradient of the image (GRMSE) [19] between the CBCT and deblurred images. Contrast enhancement of the lesions is also evaluated using the contrast-to-noise ratio (CNR). The effectiveness of the proposed deblurring method is analyzed by comparing the restored frequency components between the CBCT and deblurred images. Experiments with 3D breast volume datasets demonstrate that our proposed network achieves excellent deblurring compared to the network described in our previous study [15].

## Methods

### Data preparation

In Phase 1 training, generated 100 CBCT and DBT volume pairs using the characteristics of clinical mammograms [20, 21] were divided with a ratio of 1:1:3 for training, validation, and testing set, respectively. The testing set used in Phase 1 was divided with a ratio of 1:1:1 for training, validation, and testing set, respectively, further being used to train Phase 2 CNN.

Breast volumes were simulated using a randomly generated inverse power law noise model [22, 23]. A Gaussian noise volume of voxel size 899 × 899 × 899 pixels was generated and transformed into the frequency domain using the discrete Fourier transform (DFT). The transformed volume was multiplied with a filtering kernel (i.e., 1/*f*^3/2^, where *f* is the radial frequency in per millimeter) and transformed into the spatial domain via the inverse DFT [20] to obtain the actual breast statistics. Note that the zero frequency value of the filter was designated as twice the first non-zero frequency component to prevent an infinite value at zero frequency [24]. To avoid the wrap-around effect caused by DFT, a central spherical volume with a diameter of 450 voxels was extracted. Next, to implement a 30% volumetric glandular fraction (VGF), the voxel values were sorted in descending order. The upper (lower) 30% (70%) were assigned 0.0802 *cm*^−1^(0.0456 *cm*^−1^), corresponding to the attenuation coefficient of the glandular (adipose) tissue at an energy of 20 keV [25]. A rectangular volume with a short z-axis direction (i.e., 288 × 288 × 144) was extracted, reflecting a compressed breast volume. We generated projection data from the rectangular volume using Siddon’s algorithm [26]. The DBT image was reconstructed using 41 projection data (−20° to 20°), and the CBCT image was reconstructed using 360 projection data (−180° to 180°) based on the FDK algorithm with a Hanning-weighted ramp filter. We did not use a slice thickness (ST) filter [27] to maintain the high-frequency components [21] of the breast volume.

**Fig 2:**
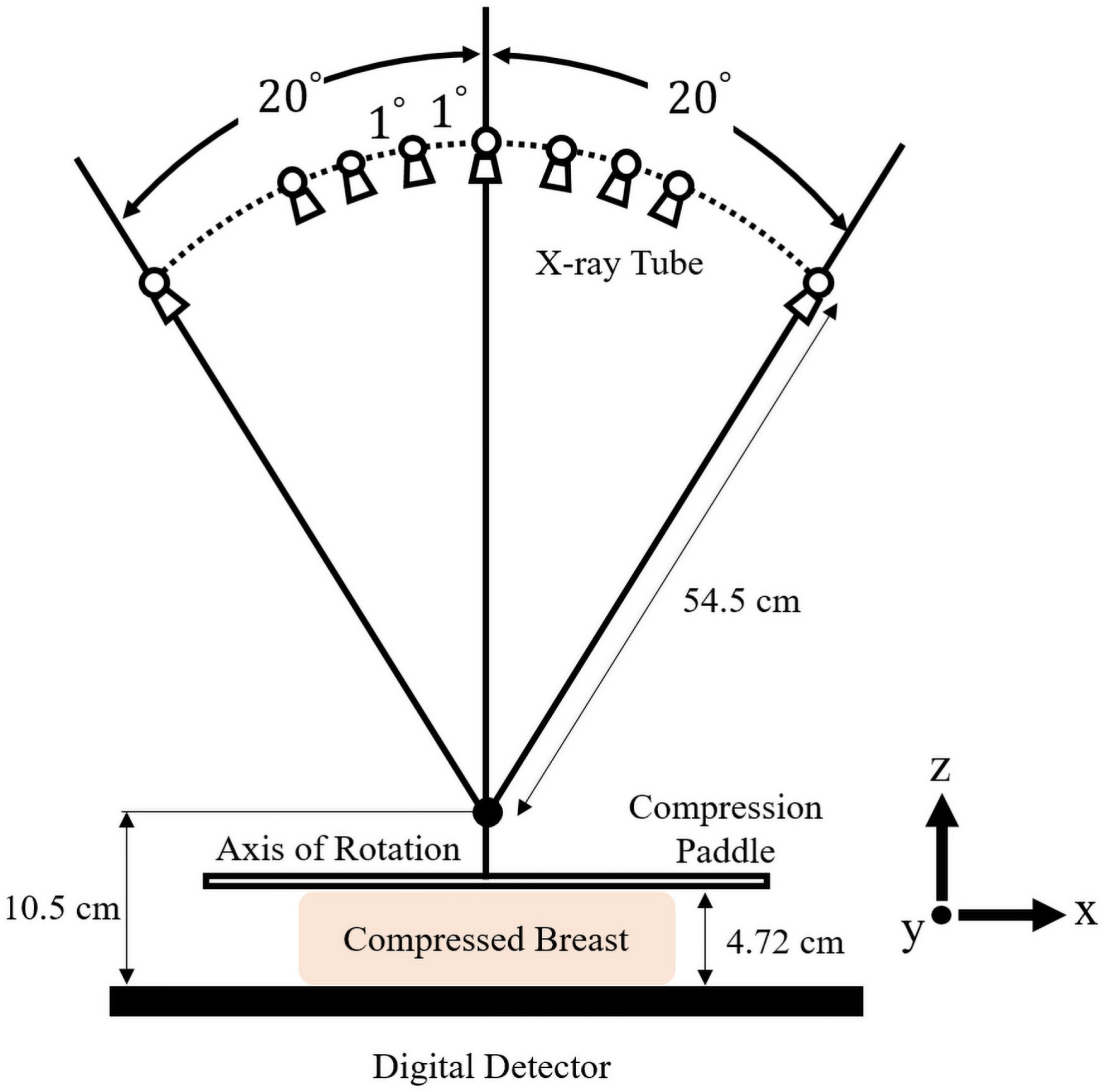
Data acquisition geometry of the DBT system.

Fig 2 illustrates the data acquisition geometry of the DBT system, and Table 1 summarizes the details of the simulation parameters. For noise simulation, quantum noise with Poisson statistics 2 × 10^5^ incident photons per detector cell, which is equivalent to the dose level of 1.6 mGy for a 4 *cm* breast with 20 KeV energy, was added to the projection data. The dose level is similar to the exposure level measured in the work of Zeng et al [28]. The total flux was matched for the DBT and CBCT data acquisition systems. Breast tissue near the volume center was replaced by a 2 *mm* or 4 *mm* diameter spherical lesion in 40 test volumes to examine the generalization performance of the trained CNN. The attenuation coefficient of the lesion was 0.0844 *cm*^−1^, corresponding to 20 keV [25] energy. To evaluate the generalized performance for different background structures, we applied the trained CNN model to deblur 15% VGF DBT images, as the 15% VGF represents the median value of women’s VGF statistics [29].

**Table 1:** Simulation parameters.

Parameters	CBCT	DBT
Source to iso-center distance	545 *mm*	
Detector to iso-center distance	105 *mm*	
Data acquisition angle	−180° ∼ 180°	−20° ∼ 20°
Number of views	360	41
Detector cell size	0.125 × 0.125*mm*^2^	
Detector array size	450 × 450	
Reconstructed volume size	47.2 × 47.2 × 47.2*mm*^3^	
Reconstructed voxel size	0.105 × 0.105 × 0.105*mm*^3^	
VGF	30% (Training and testset)	
	15% (Generalization testset)	
Number of incident X-ray photons	2 × 10^5^/*Number of views*	
Reconstruction algorithm	FDK	

### Two-phase CNN architecture

**Fig 3:**
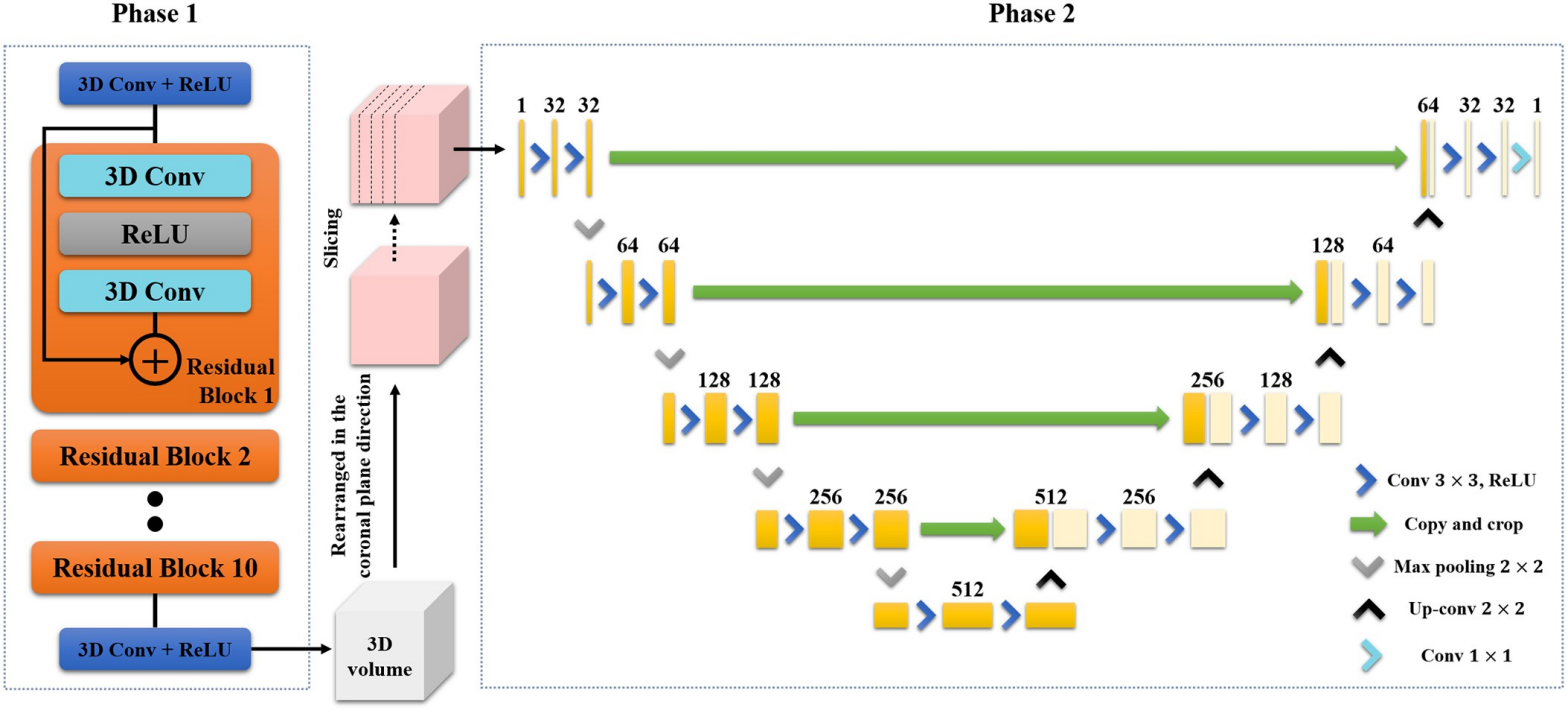
Architecture of the proposed CNN. The proposed CNN is composed of two phase. Phase 1 is composed of several residual blocks. The 2D slice of the coronal plane of the Phase 1 output volume is the input of Phase 2. Phase 2 has the structure of U-Net and it yields the final output of the proposed CNN.

Our proposed method was motivated by the model-stacking approach. Although the training time is relatively longer than that required for a single network training, previous studies [30, 31] have shown that model-stacking demonstrates better performance in terms of accuracy in medical image segmentation and classification. We focused on the fact that our target dataset is a 3D DBT volume dataset, which has similar visual patterns across the training dataset as other fine-grained datasets. Inspired by the success of the model-stacking approach in prediction tasks on fine-grained datasets, we employed it in our artifact compensation procedure on the 3D DBT volume. Various studies [32, 33] have confirmed the advantages of model-stacking for more accurate prediction and reliable estimation than the single network model for the same number of filters. Owing to these benefits, we adopted a two-phase learning-based approach. The proposed CNN architecture is presented in Fig 3.

The depth of the CNN model must be increased to increase the modeling power of Phase 1. In this case, however, the training became more difficult because of the gradient vanishing problem [34, 35]. Therefore, the residual network was adopted to increase the model capacity to mitigate the gradient vanishing problem [36, 37]. Phase 1 has several residual network building blocks [38]. Each residual block comprises of two different steps. First, an input passes through the convolutional layer, rectified linear unit (ReLU) layer, and an additional convolutional layer. Second, the input and the output of the first step are added.

Phase 2 was designed to render the output of Phase 1 more accurately by learning the texture of the breast tissue and reducing any remaining blurring artifacts in the coronal plane. As the PSF in the coronal plane is particularly wide, the most severe blurring artifacts are produced here compared to other image planes. The U-Net was adopted to reflect this during the deblurring procedure because it is known to have a wide receptive field. As indicated in Phase 2 of Fig 3, the number of filters is doubled when the feature map passes through the max-pooling layer, whereas the number of filters is halved when the feature map passes through the upsampling layer.

### Loss function

In Phase 1, we used the mean absolute error (MAE) as a loss function for the relatively good sharpness of the output image [39]. The MAE loss function is defined as follows:

$$\begin{array}{c} \begin{array}{c} L_{MAE}=\frac{1}{w\times h}{∥G_{1}\left(z\right)-x∥}_{1}, \end{array} \end{array}$$ (2)

where, *G*_1_ is the Phase 1 CNN, *x* is the CBCT image, *z* is the input DBT image reconstructed using the FDK algorithm, and *w* and *h* are the width and height of the input DBT image, respectively.

**Algorithm 1 Optimization procedure of PL-MAE**.

**Require**: Set hyperparameters, *α* = 5 × 10^−3^, *β*_1_ = 0.9, *β*_2_ = 0.999, λ_2_ = 0.05, the number of total epochs, *N*_*epoch*_ = 100, the batch size *n* = 2, and patch size of 48 × 48 × 48.

**Require**: Initial *G*_1_ (i.e., Phase 1) parameters *φ*_0_, initial *G*_2_ (i.e., Phase 2) parameters *θ*_0_

**Require**: Load pretrained VGG-16 network parameters

1: **for** *epoch* = 0, …, *N*_*epoch*_ **do**

2:  Sample a batch of DBT image patches ${\left\{z^{\left(i\right)}\right\}}_{i=1}^{n}$ and corresponding CBCT patches ${\left\{x^{\left(i\right)}\right\}}_{i=1}^{n}$

3:  **for** *i* = 1, …, *n* **do**

4:   *L*^(*i*)^(*G*_1_)←*L*_*MAE*_(*z*^(*i*)^, *x*^(*i*)^)

5:  **end for**

6:  Update $G_{1},\phi \leftarrow Adam\left({▽}_{\phi }\frac{1}{n}\sum_{n}^{i=1}L^{\left(i\right)}\left(G_{1}\right),\phi ,\alpha ,\beta_{1},\beta_{2}\right)$

7: **end for**

8: Aggregate *G*_1_(*z*;*φ*) in the form of 3D volume $\hat{z}$, aggregate *G*_1_(*x*;*φ*) in the form of 3D volume $\hat{x}$

9: Divide $\hat{z}$ into coronal slices $\tilde{z}$, divide $\hat{x}$ into coronal slices $\tilde{x}$

10: **for** *epoch* = 0, …, *N*_*epoch*_ **do**

11:  Sample a batch of ${\left\{{\tilde{z}}^{\left(i\right)}\right\}}_{i=1}^{n}$ and ${\left\{{\tilde{x}}^{\left(i\right)}\right\}}_{i=1}^{n}$

12:  **for** *i* = 1, …, *n* **do**

13:   $L^{\left(i\right)}\left(G_{2}\right)\leftarrow \lambda_{2}L_{PL}\left({\tilde{z}}^{\left(i\right)},{\tilde{x}}^{\left(i\right)}\right)+L_{MAE}\left({\tilde{z}}^{\left(i\right)},{\tilde{x}}^{\left(i\right)}\right)$

14:  **end for**

15:  Update $G_{2},\theta \leftarrow Adam\left({▽}_{\theta }\frac{1}{n}\sum_{n}^{i=1}L^{\left(i\right)}\left(G_{2}\right),\theta ,\alpha ,\beta_{1},\beta_{2}\right)$

16: **end for**

After deblurring the DBT images in Phase 1, further deblurring was performed in Phase 2. To investigate the effect of the loss function on breast tissue restoration, we used the pixel-based loss function (i.e., MAE), adversarial loss function with MAE (AL-MAE), and perception-based loss function with MAE (PL-MAE). For the adversarial loss function, we used the Wasserstein generative adversarial network with a gradient penalty (WGAN-GP) [17] and a discriminator of 144 × 144 PatchGAN [40]. The adversarial-based loss function is defined as follows:

$$\begin{array}{c} \begin{array}{c} L_{AL}=D\left(G_{2}\left(z\right)\right)-D\left(x\right)+\eta {\left(∥\nabla_{\hat{x}}D\left(\hat{x}\right){∥}_{2}^{2}-1\right)}^{2}, \end{array} \end{array}$$ (3)

where, *D* is a discriminator, *G*_2_ is the Phase 2 CNN, ∇ denotes the gradient, $\hat{x}=\epsilon x+\left(1-\epsilon \right)z$, and *ϵ* has the standard uniform distribution. The weighting parameter *η* was set to 0.1 following the recommendations in previous work [17].

We used the first 13 layers of the VGG-16 network [41] for the perception-based loss function, which was pretrained on the ImageNet dataset [42]. The CBCT and deblurred images of the proposed CNN model were passed through the VGG-16 network, and the outputs were used for loss calculation. The perception-based loss function is defined as follows:

$$\begin{array}{c} \begin{array}{c} L_{PL}=\frac{1}{W\times H\times C}{∥\phi (G_{2}\left(z\right))-\phi \left(x\right)∥}_{2}^{2}, \end{array} \end{array}$$ (4)

where, *W*, *H*, and *C* are the width, height, and the number of channels in the feature space, and *ϕ* is the feature extractor.

When using the adversarial-based and perception-based loss functions, MAE loss was used together to render a deblurred image that is more similar to the CBCT image. We determined the weighting values (i.e., λ_1_ and λ_2_) to minimize the loss function on the validation dataset in a search range of [0.0001 0.1]. The optimal values were 0.001 and 0.05 for λ_1_ and λ_2_ respectively. The objective functions of Phase 2 (i.e., MAE, AL-MAE, and PL-MAE loss functions) are defined as follows.

$$\begin{array}{c} L_{AL-MAE}=L_{MAE}+\lambda_{1}L_{AL}, \end{array}$$ (5)

$$\begin{array}{c} L_{PL-MAE}=L_{MAE}+\lambda_{2}L_{PL}, \end{array}$$ (6)

The definition of the MAE loss function is the same as in (2), except that *G*_1_ is replaced by *G*_2_.

### Training and test dataset

In Phase 1 training, a total of 20 CBCT and DBT volume pairs (i.e., 288 × 288 × 144) was used. Each of the volume pairs was divided into 108 non-overlapping patches of size 48 × 48 × 48; a total of 2,160 patch pairs was used during the training. After passing the DBT image patches through the trained Phase 1, the output patches were aggregated in the form of breast volume and separated into the 288 coronal plane slices of size 288 × 144. These output slices of Phase 1 and the corresponding CBCT slices were used for Phase 2 training. Since we used 20 volumes during the Phase 2 training, a total of 5,760 slice pairs was used.

### Model and implementation details

**Fig 4:**
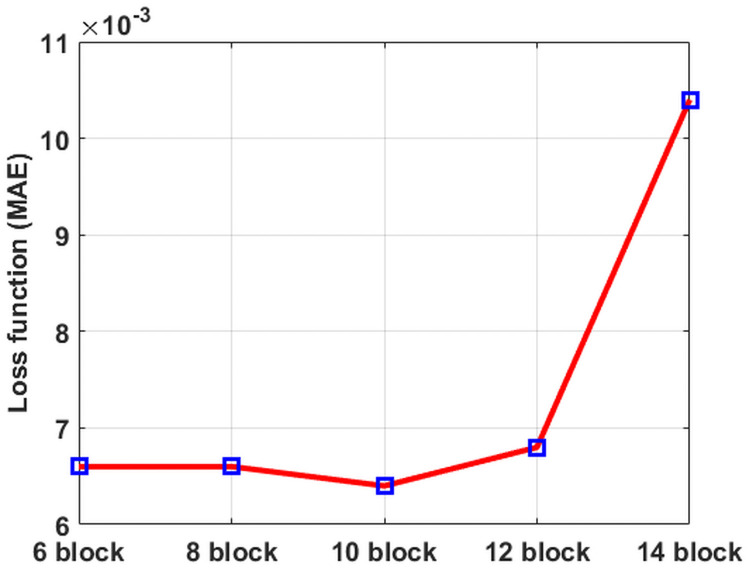
The influence of different residual network building blocks in Phase 1.

**Fig 5:**
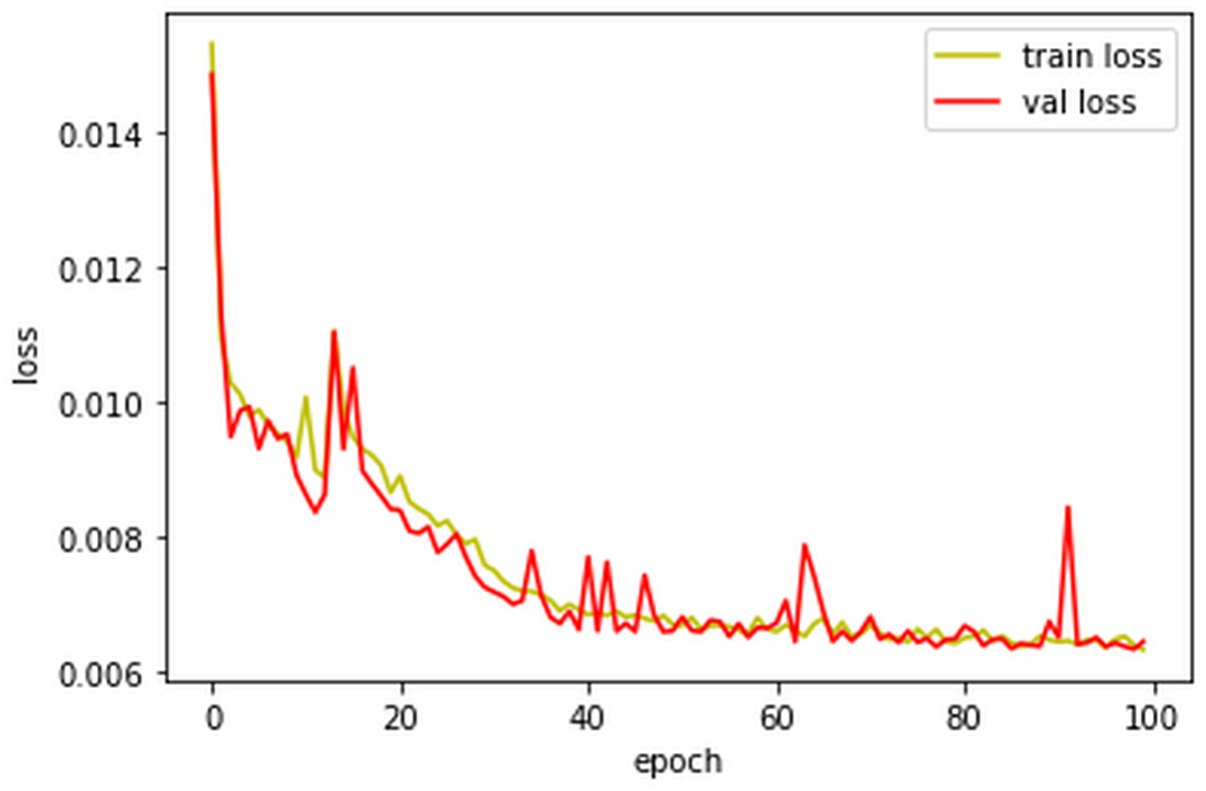
The training and validation loss of the ten-block CNN (i.e., Phase 1) with each training epoch.

In Phase 1, the network was composed of the residual network building blocks, and all convolutional layers have 40 filters of 3 × 3 × 3 size with a stride 1. The number of filters was selected experimentally to achieve the best performance without sacrificing training efficiency. We attached these results in the supplementary material. We evaluated different network depths by adjusting the number of residual network building blocks to 6, 8, 10, 12, and 14 blocks. The network with 10 residual network building blocks is superior to the others, as depicted in Fig 4. Furthermore, Fig 5 demonstrates the capability of the 10-block CNN on the validation dataset. Thus, we selected 10 residual network building blocks for the network design of Phase 1.

In Phase 2, to restore the fine texture of breast tissue, we used U-Net structures with 32 filters of size 3 × 3 with a stride of 1, which have a 140 × 140 wide receptive field size covering the PSF of the coronal plane. The PSF of the coronal plane has an elongated shape spanning a 60-pixel length.

In Phase 1, the network was trained using the adaptive moment estimation (Adam) optimizer [43] with a batch size of 2 due to the memory issue. We excluded the batch normalization layer, as our network is stably trained without the batch normalization layer. The training efficiency with and without batch normalization is compared in the supplementary material. We trained the network using 100 epochs by setting the Adam optimizer’s exponential decay rates for the first and second moment (i.e., *β*_1_ and *β*_2_) estimates to 0.9 and 0.999, respectively, as recommended in the previous study [43]. The learning rate (i.e., *α*) was 5 × 10^−3^, which was found experimentally in the range [0.0001 0.01].

In Phase 2, we used the same Adam optimizer and hyperparameters as in Phase 1 and observed that the CNN with all proposed loss functions converged stably within 100 epochs in each phase. Convergence required about 8 h each using the Keras library on a system with an Nvidia Titan XP (Pascal) 12 GB GPU and Intel (R) Core (TM) i7–6700 3.40 GHz processor.

### Performance evaluation

#### Pixel-based evaluation

The means of the 2D MSE and 2D GRMSE were used to evaluate the similarities between the CBCT and deblurred images. The mean of the 2D MSE is calculated as follows:

$$\begin{array}{c} \begin{array}{c} {MSE}_{mean}\left(y,\tilde{y}\right)=\frac{1}{mn}\sum_{i=1}^{m}\sum_{j=1}^{n}(y_{ij}-{\tilde{y}}_{ij}{)}^{2}, \end{array} \end{array}$$ (7)

where *y*_*ij*_ is the *j*^*th*^ pixel of the central slice image of *i*^*th*^ CBCT volume, ${\tilde{y}}_{ij}$ is the *j*^*th*^ pixel of the central slice image of the *i*^*th*^ deblurred volume, *m* is the number of images, and *n* is the number of pixels in the image.

For the subjective visual assessment [19], we used the mean of the 2D GRMSE, defined as follows:

$$\begin{array}{c} \begin{array}{c} {GRMSE}_{mean}\left(y,\tilde{y}\right)=\frac{1}{m}\sum_{i=1}^{m}\sqrt{\frac{\sum_{j=1}^{n}{\left(O\left(y_{ij}\right)-O\left({\tilde{y}}_{ij}\right)\right)}^{2}}{n}}, \end{array} \end{array}$$ (8)

where we used the intermediate operator *O* as a gradient function. The mean of the 2D MSE and 2D GRMSE between the CBCT and deblurred images were compared in the axial, coronal, and sagittal planes.

#### Lesion contrast

The CNR was calculated for the images with 4 *mm* lesions inserted to evaluate the contrast improvement of lesions against the background in the deblurred image. The CNR is strongly associated with the reader preference score for lesion contrast [44]. We extracted the central slice of the breast volume to calculate the CNR. In the extracted slice, the circular-shaped lesion was set as the foreground, and the outer part of the lesion was set as the background. The mean of CNR is calculated as follows:

$$\begin{array}{c} \begin{array}{c} {CNR}_{mean}=\frac{1}{m}\sum_{i=1}^{m}\frac{\left|u_{f}\left(y_{i}\right)-u_{b}\left(y_{i}\right)\right|}{\sqrt{(\sigma_{f}^{2}\left(y_{i}\right)+\sigma_{b}^{2}\left(y_{i}\right))/2}}, \end{array} \end{array}$$ (9)

where *y*_*i*_ is the central slice image of *i*^*th*^ CBCT volume, ${\tilde{y}}_{i}$ is the central slice image of the *i*^*th*^ deblurred volume, *u*_*b*_(*u*_*f*_) is the mean CT number outside (inside) the mass lesion, and *σ*_*b*_(*σ*_*f*_) is the standard deviation outside (inside) the mass lesion.

#### Frequency domain analysis

To evaluate the ability of the CNN to fill in the missing data of the DBT image in the frequency domain, we examined the frequency responses for the axial, coronal, and sagittal planes. We extracted the central slice in each direction from 20 independently generated breast volumes. Then, the extracted images were 2D Fourier transformed and its absolute values were averaged. We displayed them on the log scale. The MSE values between the 2D FFTs of the CBCT and deblurred images were compared. The central vertical profiles of each 2D frequency response were also compared, as this area contains the most missing data in the DBT images.

## Results

**Fig 6:**
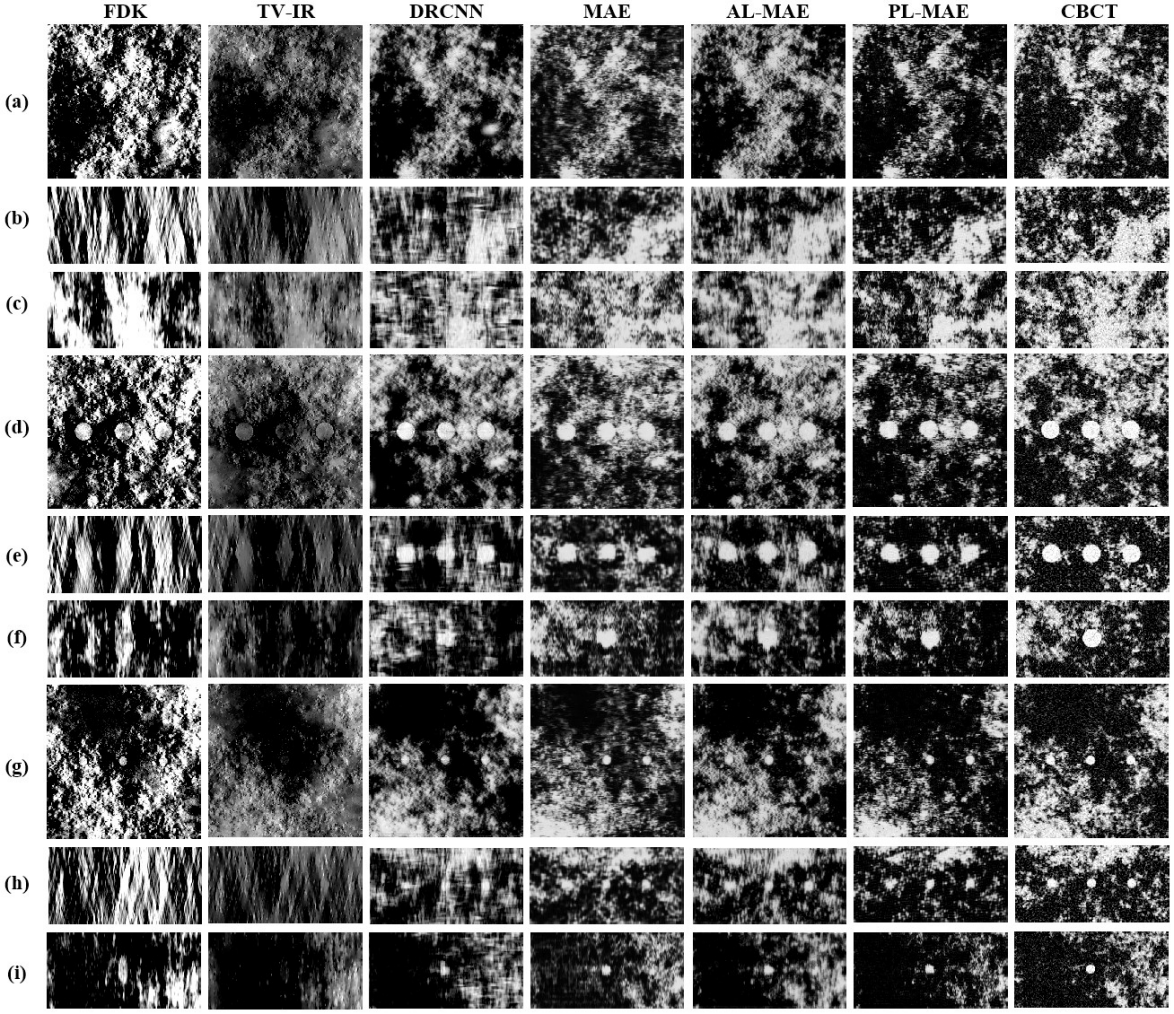
Results using 30% VGF DBT images. DBT images reconstructed with FDK and TV-IR, deblurred images by DRCNN, deblurred images by the proposed method with MAE, AL-MAE, and PL-MAE loss functions, and CBCT images (from left to right). Images without mass lesion for (a) axial, (b) coronal, and (c) sagittal planes; images containing 4 *mm* lesions for (d) axial, (e) coronal, and (f) sagittal planes, and images containing 2 *mm* lesions for (g) axial, (h) coronal, and (i) sagittal planes. The display window is [0.0456 0.0844] in *cm*^−1^.

We compared the proposed two-phase learning-based scheme with the FDK algorithm, total-variation iterative reconstruction with GP-BB (TV-IR), and DRCNN [15]. In TV-IR method, we applied the algorithm by setting the iteration number to 100 and regularization parameter (i.e., λ) to 5 × 10^−4^. In DRCNN method, we trained the network using 100 epochs by setting the *β*_1_ and *β*_2_ estimates to 0.9 and 0.999, respectively. The learning rate was 1 × 10^−3^, which was found experimentally in the range [0.0001 0.01]. Fig 6 illustrates the DBT images reconstructed using the FDK algorithm and TV-IR, DRCNN, CNN-based deblurred images with different loss functions, and CBCT images. In Fig 6(a)–6(c), we observe that severe blurring artifacts in the DBT image are reduced in all planes using the proposed method. In particular, the coronal and sagittal planes of the DBT image contain very severe blurring artifacts due to the limited range of data acquisition angles, and it is difficult to recognize the original structures compared to the axial plane. We also observed that GP-BB has a low lesion contrast compared with the FDK algorithm, although it could enhance the edge. In the image deblurred using DRCNN, the deblurring is not properly performed in the coronal and sagittal planes. However, the proposed deblurring method recovers the original structures of these planes reliably with notably improved image quality.

We also observed that different loss functions produce different textures in the deblurred images. Compared with the CBCT images, the deblurred images with MAE loss functions introduce slight blurs, reflected as reduced image noise. In addition, we observed that the proposed method using AL-MAE loss overestimated the original structures and amplified the noise. Using the proposed method with WGAN-GP loss to restore extensive missing data in DBT images would not be appropriate due to the amplification of high-frequency components. However, the deblurred images with PL-MAE loss functions exhibit textures more similar to the CBCT images due to their ability to preserve feature information via the perception-based loss functions. Overall, the image sharpness is preserved well in the deblurred images with MAE and PL-MAE loss functions.

Table 2 summarizes the means of the 2D MSE and 2D GRMSE between the CBCT and deblurred images for different loss functions. A smaller value indicates better performance in the MSE and GRMSE. In the DBT image, we only used the axial plane for pixel-based evaluations because the other planes do not contain any useful information due to the severe blurring. The quantitative results confirm our observation in Fig 6, implying that the proposed method achieves excellent deblurring performance comparable to or better than that of the DRCNN. Compared with other loss functions, the deblurred image by MAE provides slightly better scores in terms of the MSE. This result may be attributable to the generated anatomical background image being relatively piecewise linear, thus rendering the MAE loss function more appropriate for these metrics. As GRMSE reflects perceptual characteristics, the deblurred image using PL-MAE provides better results in the GRMSE evaluation.

**Table 2:** Pixel-based evaluation with 30% VGF DBT images. MSE and GRMSE results of the DBT images reconstructed with FDK and TV-IR, deblurred images by DRCNN, deblurred images by the proposed method with MAE, AL-MAE, and PL-MAE. (mean±standard deviation).

	MSE (×10^−5^)			GRMSE (×10^−2^)		
Method	Axial	Coronal	Sagittal	Axial	Coronal	Sagittal
DRCNN	9.67 ± 1.01	10.51 ± 2.20	10.21 ± 2.06	0.92 ± 0.03	0.87 ± 0.07	0.77 ± 0.06
MAE	**7.09 ± 0.78**	**8.28 ± 1.32**	**7.56 ± 1.20**	**0.59 ± 0.01**	0.59 ± 0.01	**0.64 ± 0.01**
AL-MAE	8.72 ± 3.40	8.51 ± 1.82	11.48 ± 0.12	0.65 ± 0.15	0.56 ± 0.01	0.73 ± 0.20
PL-MAE	8.90 ± 1.30	10.02 ± 2.15	9.62 ± 2.06	**0.59 ± 0.01**	**0.55 ± 0.01**	**0.64 ± 0.02**
FDK	41.09 ± 5.56			1.07 ± 0.06		
TV-IR	11.04 ± 2.39			0.61 ± 0.01		

Fig 6(d)–6(i) displays the DBT images reconstructed by FDK algorithm and TV-IR, deblurred images, and CBCT images with the presence of 4 *mm* and 2 *mm* diameter lesions. Three lesions were included along the *x*-direction to examine how well the proposed method can recover spatially varying blurring artifacts in DBT images. It is challenging to identify the lesions in the coronal and sagittal planes in the DBT image due to the severe blurring artifacts. In the image deblurred by the DRCNN, the shapes of lesions are distorted. However, the proposed method restores the original lesion shapes more effectively. In particular, the lesion detectability in the coronal and sagittal planes is superior to the FDK algorithm, TV-IR, and DRCNN. Despite the powerful deblurring performance, we observed that the boundaries of 4 *mm* and 2 *mm* lesions are not recovered well in the coronal and sagittal planes compared to the axial plane. It appears that the proposed CNN experiences difficulties in filling extensive missing data in the coronal and sagittal planes compared to the axial plane.

Table 3 summarizes the CNR of each plane for the 4 *mm* lesions. In the 4 *mm* lesions, the deblurred axial plane image achieves significantly improved CNR performance, which is 2.84 times higher than that of the original DBT image reconstructed using the FDK algorithm. Even coronal and sagittal plane images exhibit a much higher CNR than DRCNN images. While all loss functions provide similar improvements in the CNR, the PL-MAE achieves a relatively higher CNR over all planes than other loss functions because the PL-MAE compromises the sharpness and textures of the original image more effectively for this task. Through the CNR results, the CNR of the axial plane has a relatively high value compared with the other planes. As the missing data in the axial plane is smaller than in the other planes, the lesion contrast improvement of the proposed method seems better.

**Table 3:** Lesion contrast evaluation with 30% VGF DBT images. CNR results of the DBT images reconstructed with FDK and TV-IR, deblurred images by DRCNN, deblurred images by the proposed method with MAE, AL-MAE, and PL-MAE. (mean±standard deviation).

	Contrast-to-Noise Ratio		
Method	Axial	Coronal	Sagittal
DRCNN	2.25 ± 0.54	2.01 ± 0.57	1.98 ± 0.65
MAE	2.91 ± 0.79	2.42 ± 0.76	2.67 ± 0.73
AL-MAE	2.17 ± 0.81	2.06 ± 0.66	2.04 ± 0.82
PL-MAE	**3.24 ± 0.75**	**2.98 ±1.05**	**3.19 ± 1.00**
FDK	1.14 ± 0.57		
TV-IR	0.65 ± 0.42		

**Fig 7:**
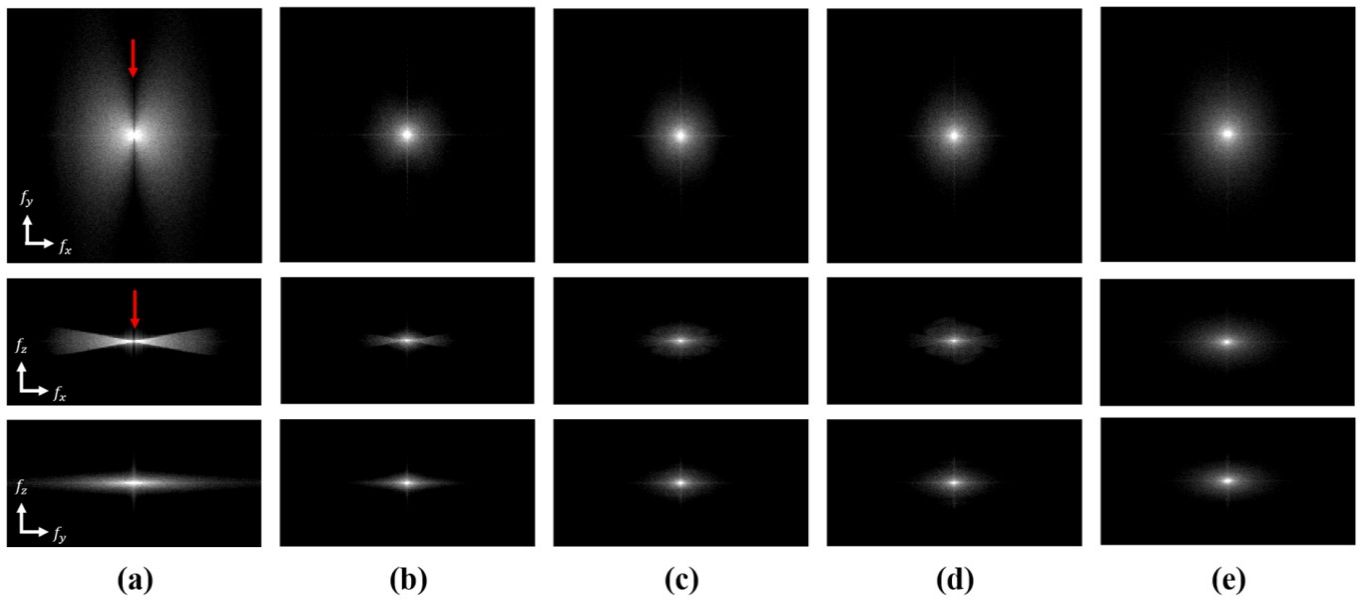
Frequency domain analysis. Frequency responses of DBT images using FDK, deblurred images, and CBCT images for *f*_*x*_-*f*_*y*_ plane (Top), *f*_*x*_-*f*_*z*_ plane (middle), and *f*_*y*_-*f*_*z*_ plane (bottom). (a) DBT images with FDK reconstruction, (b) deblurred images after Phase 1 and deblurred images after Phase 2 with (c) MAE, (d) PL-MAE, and (e) CBCT images. The display window is [1 4]. The red arrows indicate the missing data regions in the DBT images.

**Fig 8:**
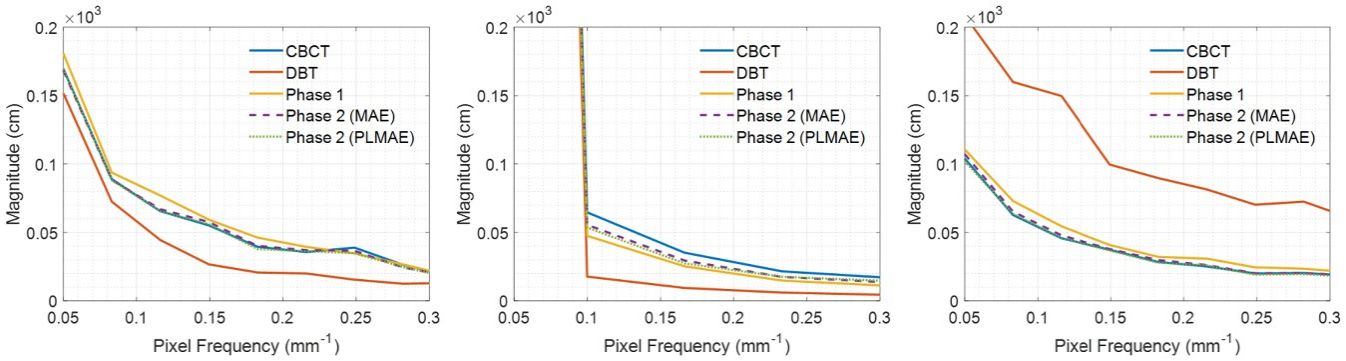
Central vertical profiles of the frequency domain. Central vertical profiles of Fig 7 for (a) *f*_*x*_-*f*_*y*_, (b) *f*_*x*_-*f*_*z*_, and (c) *f*_*y*_-*f*_*z*_ planes. Note that *f* is the pixel frequency in *mm*^−1^.

The 2D frequency responses of the deblurred images from Phases 1 and 2 were calculated, as listed in Fig 7, to analyze the restoring power of the proposed method in each phase. We selected the MAE (PL-MAE) loss for this comparison because it yielded the highest performance in the pixel-based evaluation (lesion contrast) with the CBCT images. As expected, the DBT image contains many missing data points due to the limited data acquisition angle, as depicted in Fig 7(a). However, most of the missing data are appropriately filled in by the proposed method. Note that the high-frequency components are observed in the DBT image because the ST filter is not applied. When we used the ST filter, the high-frequency components are reduced, which is common in many breast tomosynthesis imaging cases. The DBT images with a ST filter are included in the supplementary material. Table 4 summarizes the MSE between the 2D FFTs of the CBCT and deblurred images. The PL-MAE achieves a relatively low MSE value in all planes, demonstrating the effectiveness of using the perception-based loss function to fill in the missing data in the frequency domain. Fig 8 compares the central vertical profiles in Fig 7 for the CBCT, DBT, and deblurred images with MAE and PL-MAE loss functions. The proposed method sequentially restores the missing frequency components as the DBT image is processed through Phases 1 and 2. As we intended, Phase 1 performs the initial deblurring to fill in the missing data of the DBT image, as presented in Fig 7(b), but small differences in the CBCT image are still observed, as presented in Fig 8. The image sharpness is restored further by Phase 2, producing improved similarity between the CBCT and deblurred images, as indicated in Fig 8.

**Table 4:** MSE between the 2D FFTs of the CBCT and deblurred images by the proposed method with MAE and PL-MAE.

	MSE in the frequency domain		
Method	Axial	Coronal	Sagittal
Phase 1	0.82	1.31	1.12
Phase 2 (MAE)	0.51	0.59	0.34
Phase 2 (PL-MAE)	**0.42**	**0.57**	**0.29**
FDK	12.21	11.91	6.19

**Fig 9:**
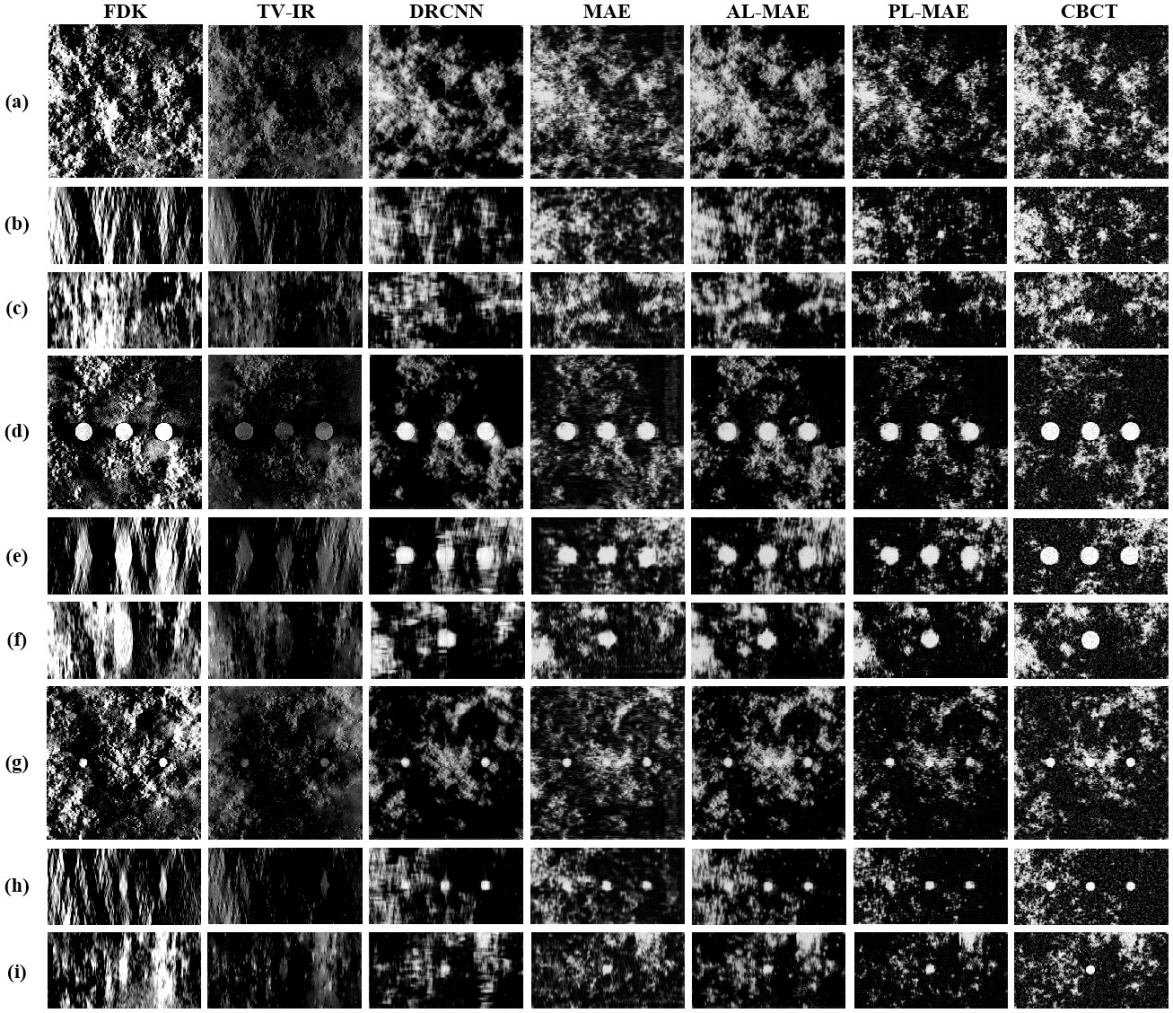
Results using 15% VGF DBT images. VGF 15% DBT images reconstructed with FDK and TV-IR, deblurred images by DRCNN, deblurred images by the proposed method with MAE, AL-MAE, and PL-MAE loss functions, and CBCT images (from left to right). Images without mass lesion for (a) axial, (b) coronal, and (c) sagittal planes; images containing 4 *mm* lesions for (d) axial, (e) coronal, and (f) sagittal planes, and images containing 2 *mm* lesions for (g) axial, (h) coronal, and (i) sagittal planes. The display window is [0.0456 0.0844] in *cm*^−1^.

The proposed method’s generalization performance is tested using 15% VGF data, and the corresponding deblurred images are illustrated in Fig 9. The results demonstrate that the proposed CNN is still effective for reducing blurring artifacts and exhibits robust characteristics, even for unseen data. The results of the quantitative evaluation are summarized in Tables 5 and 6. The proposed CNN using different loss functions demonstrated better results than DRCNN over all planes, even for unseen data through these results. In particular, the CNN using AL-MAE exhibits good performance for generalization tests represented by the MSE results. In contrast, the CNN using PL-MAE still produces the best score using the GRMSE.

**Table 5:** Pixel-based evaluation with 15% VGF DBT images. MSE and GRMSE results of the DBT images reconstructed with FDK and TV-IR, deblurred images by DRCNN, deblurred images by the proposed method with MAE, AL-MAE, and PL-MAE in generalization testset. (mean±standard deviation).

	MSE (×10^−5^)			GRMSE (×10^−2^)		
Method	Axial	Coronal	Sagittal	Axial	Coronal	Sagittal
DRCNN	6.33 ± 1.41	8.89 ± 2.50	7.60 ± 1.89	0.81 ± 0.04	0.69 ± 0.06	0.66 ± 0.04
MAE	4.62 ± 1.23	5.06 ± 1.23	**4.93 ± 1.45**	0.57 ± 0.01	0.56 ± 0.02	0.62 ± 0.02
AL-MAE	**4.47 ± 1.32**	**4.63 ± 1.35**	6.16 ± 4.91	0.60 ± 0.02	0.56 ± 0.06	0.69 ± 0.01
PL-MAE	5.10 ± 1.80	5.26 ± 1.89	5.63 ± 2.10	**0.56 ± 0.02**	**0.51 ± 0.02**	**0.60 ± 0.02**
FDK	23.47 ± 7.98			0.85 ± 0.10		
TV-IR	7.10 ± 2.67			**0.56 ± 0.02**		

**Table 6:** Lesion contrast evaluation with 15% VGF DBT images. CNR results of the DBT images reconstructed with FDK and TV-IR, deblurred images by DRCNN, deblurred images by the proposed method with MAE, AL-MAE, and PL-MAE in generalization testset. (mean±standard deviation).

	CNR		
Method	Axial	Coronal	Sagittal
DRCNN	3.22 ± 0.49	2.98 ± 0.61	3.22 ± 0.86
MAE	4.27 ± 0.85	3.64 ± 0.84	3.32 ± 0.77
AL-MAE	1.94 ± 1.12	2.97 ± 1.12	1.96 ± 1.08
PL-MAE	**4.67 ± 0.92**	**4.25 ± 0.80**	**3.84 ± 0.83**
FDK	1.61 ± 0.40		
TV-IR	1.07 ± 0.40		

Based on the different values of VGF, we compared the relative improvements from the MSE, GRMSE, and CNR based on the data from the axial plane of the DBT image reconstructed using the FDK algorithm. For the 15% (30%) VGF dataset, the MSE and GRMSE decreased by 81.0% (82.8%) and 34.1% (44.9%), respectively, compared with the DBT image, and the CNR increased by 191.2% (183.4%) compared with the DBT image.

## Discussion and conclusion

In this study, we reduced the blurring artifacts in the DBT images using a two-phase learning-based CNN and evaluated the image quality using the MSE, GRMSE, and CNR. Although the simulated lesions included in the DBT image were slightly distorted, images deblurred by the proposed method achieved a higher CNR compared with the conventional method. We also demonstrated that the proposed method could reduce the blurring artifacts for unseen data, which was tested using data obtained based on different VGF values.

Given the limited access to actual breast CT volumes, we validated the proposed method using 3D volume data generated using a computer simulation. Further validation of the proposed method using clinically available DBT image datasets could be an interesting future research topic. We generated the training data pair by generating the DBT and CBCT volume using a computer simulation in this work. In actual clinical situations, acquiring such paired data would not be feasible. In this case, the DBT image can be generated by conducting a forward projection of the CBCT volume by reflecting the data acquisition geometry of the DBT system.

We specifically aimed to achieve digital tomosynthesis image deblurring in anatomical backgrounds, but the proposed two-phase CNN structure could also be applied to deblur other digital tomosynthesis images, such as chest images. The publicly available clinical CBCT chest data provided by the NIH clinical center was used to verify that the proposed CNN is effective for other types of clinical data as well. We observed that the MSE of the axial plane with the proposed method was reduced by 60% compared with the digital tomosynthesis image. We believe further improvements can be achieved using an optimized network structure and training strategy for the chest dataset, which is a topic for future research. We attached these results in the supplementary material.

We used breast volumes with 30% VGF for training and testing the model. In the previous study [29], it was reported that 80% of women have a VGF lower than 27%, and 95% have a VGF below 45%. Although the VGF is a little higher, breast volumes with a 30% VGF were generated to verify that the proposed CNN could reduce blurring artifacts under harsh conditions in which deblurring may be difficult. To examine the generalization performance of the proposed algorithm, we generated a 30% VGF DBT volume acquired over the ranges of −40° to 40° and −10° to 10° for the same breast volume. These two volumes were deblurred using the CNN, pretrained with the DBT volume acquired over the range of −20° to 20° and the corresponding CBCT volume pair. The generalization performance is much better for a larger data acquisition angle (i.e., −40° to 40°). Because the primary role of the proposed method is to fill in the missing data of DBT volume in frequency space (or equivalently, deblurring in image space), the generalization performance of the CNN for the DBT volumes acquired over a −10° to 10° data acquisition angle is worse because it contains much more missing data in frequency space. We attached these results in the supplementary material.

We adopted U-Net in phase 2, because it contains a large receptive field size to cover the length of PSF in the DBT system, which is a key aspect of the proposed method. When we used REDCNN [45] or ResNet [38] in phase 2, the performance of the deblurring was not effective compared to the case of U-Net. Detailed results are included in the supplementary material.

For further validation of the proposed method, the PSF deblurring method based on iterative blind deconvolution (i.e., PSF deblur) [13] was compared with the proposed method (i.e., PL-MAE). The image deblurred by the PSF deblur method slightly increased the CNR of 4 *mm* lesions compared to the image reconstructed by FDK, similar to the result of the previous study [13]. However, the MSE and GRMSE between the PSF deblurred image and the reference image were increased compared to the image reconstructed by the FDK due to the increased noise level. These results demonstrate that our proposed method showed a higher performance than the PSF deblur method. The following results are shown in the supplementary material.

In this study, we trained the proposed CNN using the MAE, AL-MAE, and PL-MAE loss functions. All these loss functions exhibited verifiable image quality improvement compared to the DRCNN and the FDK algorithm reconstruction methods. In particular, the PL-MAE loss function exhibited the best deblurring performance in the results for the GRMSE, CNR, and frequency domain analysis. Because previous works [46–50] have reported that adversarial loss effectively recovers missing data, we also used the WGAN-GP as a loss function to determine the performance of the proposed method. However, the deblurring performance of WGAN-GP was worse than that of the MAE and PL-MAE. We conjecture that WGAN-GP is not effective for filling in extensive missing data, as is the case for the DBT system.

We used ±20° data acquisition, which falls within the range of data acquisition angle (i.e., [±7.5° ±25°]) of the commercialized DBT systems [51]. Depending on the imaging applications, digital tomosynthesis systems use different data acquisition angles (e.g., ±25° for scaphoid [52], ±45° for head and neck [3], and ±102.5° for dental [4]), producing fewer blurring artifacts compared to the current work. Extending the proposed method to different angle digital tomosynthesis imaging systems and different background structures would be interesting for future research.

In this study, the proposed deblurring method was tested only on FDK reconstructed DBT images. However, the proposed method can also be used for any practical reconstruction as long as the training data pair can be acquired; when DBT images are reconstructed with different apodization filters, the network can be separately trained for each apodization filters. Moreover, using transfer learning [53] could be an additional solution when only a limited amount of training dataset can be acquired.

In conclusion, we proposed the two-phase learning-based 3D deblurring technique considering the wide PSF of the DBT system. We quantitatively analyzed the deblurring results using quantitative evaluation (i.e., MSE, GRMSE, and CNR). The results reveal that the proposed method performs effective 3D deblurring and reduces the blurring artifacts effectively from the in-focus plane and other planes of the DBT image. Combining the proposed method with the DBT system would be extremely useful for computer-assisted diagnosis. External validation through experimental results will be performed in future work, as all datasets used in the experiment were generated using a computer simulation.

## Supporting information

S1 File Supplementary material includes additional implementation details and further clarification. (PDF)

## References

[1] Dobbins JT III, McAdams HP. Chest tomosynthesis: technical principles and clinical update. European journal of radiology. 2009;72(2):244–51. doi: 10.1016/j.ejrad.2009.05.054 19616909 PMC3693857

[2] Duryea J, Dobbins J III, Lynch J. Digital tomosynthesis of hand joints for arthritis assessment. Medical physics. 2003;30(3):325–33. doi: 10.1118/1.1543573 12674232

[3] Bachar G, Siewerdsen J, Daly M, Jaffray D, Irish J. Image quality and localization accuracy in C-arm tomosynthesis-guided head and neck surgery. Medical physics. 2007;34(12):4664–77. doi: 10.1118/1.2799492 18196794

[4] Ogawa K, Langlais R, McDavid W, Noujeim M, Seki K, Okano T, et al. Development of a new dental panoramic radiographic system based on a tomosynthesis method. Dentomaxillofacial Radiology. 2010;39(1):47–53. doi: 10.1259/dmfr/12999660 20089744 PMC3520410

[5] Bonafede MM, Kalra VB, Miller JD, Fajardo LL. Value analysis of digital breast tomosynthesis for breast cancer screening in a commercially-insured US population. ClinicoEconomics and outcomes research: CEOR. 2015;7:53. 25624767 10.2147/CEOR.S76167 PMC4296908

[6] Gao Y, Babb JS, Toth HK, Moy L, Heller SL. Digital breast tomosynthesis practice patterns following 2011 FDA approval: a survey of breast imaging radiologists. Academic radiology. 2017;24(8):947–53. doi: 10.1016/j.acra.2016.12.011 28188043

[7] Niklason LT, Christian BT, Niklason LE, Kopans DB, Castleberry DE, Opsahl-Ong B, et al. Digital tomosynthesis in breast imaging. Radiology. 1997;205(2):399–406. doi: 10.1148/radiology.205.2.9356620 9356620

[8] Gennaro G, Toledano A, Di Maggio C, Baldan E, Bezzon E, La Grassa M, et al. Digital breast tomosynthesis versus digital mammography: a clinical performance study. European radiology. 2010;20(7):1545–53. doi: 10.1007/s00330-009-1699-5 20033175

[9] Sechopoulos I. A review of breast tomosynthesis. Part I. The image acquisition process. Medical physics. 2013;40(1):014301. doi: 10.1118/1.4770279 23298126 PMC3548887

[10] Feldkamp LA, Davis LC, Kress JW. Practical cone-beam algorithm. Josa a. 1984;1(6):612–9. doi: 10.1364/JOSAA.1.000612

[11] Park JC, Song B, Kim JS, Park SH, Kim HK, Liu Z, et al. Fast compressed sensing-based CBCT reconstruction using Barzilai-Borwein formulation for application to on-line IGRT. Medical physics. 2012;39(3):1207–17. doi: 10.1118/1.3679865 22380351

[12] Nah S, Hyun Kim T, Mu Lee K. Deep multi-scale convolutional neural network for dynamic scene deblurring. Proceedings of the IEEE conference on computer vision and pattern recognition; 2017.

[13] Mota AM, Clarkson MJ, Almeida P, Matela N. An Enhanced Visualization of DBT Imaging Using Blind Deconvolution and Total Variation Minimization Regularization. IEEE Transactions on Medical Imaging. 2020;39(12):4094–101. doi: 10.1109/TMI.2020.3013107 32746152

[14] Fish D, Brinicombe A, Pike E, Walker J. Blind deconvolution by means of the Richardson–Lucy algorithm. JOSA A. 1995;12(1):58–65. doi: 10.1364/JOSAA.12.000058

[15] Choi Y, Shim H, Baek J. Image Quality Enhancement of Digital Breast Tomosynthesis Images by Deblurring with Deep Residual Convolutional Neural Network. 2018 IEEE Nuclear Science Symposium and Medical Imaging Conference Proceedings (NSS/MIC); 2018: IEEE.

[16] Ronneberger O, Fischer P, Brox T. U-net: Convolutional networks for biomedical image segmentation. International Conference on Medical image computing and computer-assisted intervention; 2015: Springer.

[17] Gulrajani I, Ahmed F, Arjovsky M, Dumoulin V, Courville A. Improved training of wasserstein gans. arXiv preprint arXiv:170400028. 2017.

[18] Gatys LA, Ecker AS, Bethge M. Image style transfer using convolutional neural networks. Proceedings of the IEEE conference on computer vision and pattern recognition; 2016.

[19] Rose SD, Sanchez AA, Sidky EY, Pan X. Investigating simulation-based metrics for characterizing linear iterative reconstruction in digital breast tomosynthesis. Medical physics. 2017;44(9):e279–e96. doi: 10.1002/mp.12445 28901614 PMC5604860

[20] Gong X, Glick SJ, Liu B, Vedula AA, Thacker S. A computer simulation study comparing lesion detection accuracy with digital mammography, breast tomosynthesis, and cone-beam CT breast imaging. Medical physics. 2006;33(4):1041–52. doi: 10.1118/1.2174127 16696481

[21] Richard S, Samei E. Quantitative imaging in breast tomosynthesis and CT: Comparison of detection and estimation task performance. Medical physics. 2010;37(6Part1):2627–37. doi: 10.1118/1.3429025 20632574

[22] Burgess AE, Jacobson FL, Judy PF. Human observer detection experiments with mammograms and power-law noise. Medical physics. 2001;28(4):419–37. doi: 10.1118/1.1355308 11339738

[23] Reiser I, Nishikawa RM. Task-based assessment of breast tomosynthesis: Effect of acquisition parameters and quantum noise a. Medical physics. 2010;37(4):1591–1600. doi: 10.1118/1.3357288 20443480 PMC2852443

[24] Burgess AE, Judy PF. Signal detection in power-law noise: effect of spectrum exponents. JOSA A. 2007;24(12):B52–60. doi: 10.1364/JOSAA.24.000B52 18059914

[25] Johns PC, Yaffe MJ. X-ray characterisation of normal and neoplastic breast tissues. Physics in Medicine & Biology. 1987;32(6):675. doi: 10.1088/0031-9155/32/6/002 3039542

[26] Siddon RL. Fast calculation of the exact radiological path for a three-dimensional CT array. Medical physics. 1985;12(2):252–5. doi: 10.1118/1.595715 4000088

[27] Zhou J, Zhao B, Zhao W. A computer simulation platform for the optimization of a breast tomosynthesis system. Medical physics. 2007;34(3):1098–109. doi: 10.1118/1.2558160 17441255

[28] Zeng R, Park S, Kakic P, Myers KJ. Evaluating the sensitivity of the optimization of acquisition geometry to the choice of reconstruction algorithm in digital breast tomosynthesis through a simulation study. Physics in Medicine & Biology. 2015;60(3):1259. doi: 10.1088/0031-9155/60/3/1259 25591807

[29] Yaffe M, Boone JM, Packard N, Alonzo-Proulx O, Huang SY, Peressotti C, et al. The myth of the 50–50 breast. Medical physics. 2009;36(12):5437–43. doi: 10.1118/1.3250863 20095256 PMC2787062

[30] Kim M, Yun J, Cho Y, Shin K, Jang R, Bae H-j, et al. Deep learning in medical imaging. Neurospine. 2019;16(4):657. doi: 10.14245/ns.1938396.198 31905454 PMC6945006

[31] Nguyen TT, Liew AW-C, Pham XC, Nguyen MP. A novel 2-stage combining classifier model with stacking and genetic algorithm based feature selection. International Conference on Intelligent Computing; 2014: Springer.

[32] Jarrett K, Kavukcuoglu K, Ranzato MA, LeCun Y. What is the best multi-stage architecture for object recognition?. 2009 IEEE 12th international conference on computer vision; 2009: IEEE.

[33] Graczyk M, Lasota T, Trawiński B, Trawiński K. Comparison of bagging, boosting and stacking ensembles applied to real estate appraisal. Asian conference on intelligent information and database systems; 2010: Springer.

[34] Tong T, Li G, Liu X, Gao Q. Image super-resolution using dense skip connections. Proceedings of the IEEE international conference on computer vision; 2017.

[35] Han X-H, Zheng Y, Chen Y-W. Multi-level and multi-scale spatial and spectral fusion CNN for hyperspectral image super-resolution. Proceedings of the IEEE/CVF International Conference on Computer Vision Workshops; 2019.

[36] Mao X-J, Shen C, Yang Y-B. Image restoration using very deep convolutional encoder-decoder networks with symmetric skip connections. arXiv preprint arXiv:160309056. 2016.

[37] Yue B, Fu J, Liang J. Residual recurrent neural networks for learning sequential representations. Information. 2018;9(3):56. doi: 10.3390/info9030056

[38] He K, Zhang X, Ren S, Sun J. Deep residual learning for image recognition. Proceedings of the IEEE conference on computer vision and pattern recognition; 2016.

[39] Zhao H, Gallo O, Frosio I, Kautz J. Loss functions for image restoration with neural networks. IEEE Transactions on computational imaging. 2016;3(1):47–57. doi: 10.1109/TCI.2016.2644865

[40] Isola P, Zhu J-Y, Zhou T, Efros AA. Image-to-image translation with conditional adversarial networks. Proceedings of the IEEE conference on computer vision and pattern recognition; 2017.

[41] Simonyan K, Zisserman A. Very deep convolutional networks for large-scale image recognition. arXiv preprint arXiv:14091556. 2014.

[42] Russakovsky O, Deng J, Su H, Krause J, Satheesh S, Ma S, et al. Imagenet large scale visual recognition challenge. International journal of computer vision. 2015;115(3):211–52. doi: 10.1007/s11263-015-0816-y

[43] Kingma DP, Ba J. Adam: A method for stochastic optimization. arXiv preprint arXiv:14126980. 2014.

[44] Goodsitt MM, Chan H-P, Schmitz A, Zelakiewicz S, Telang S, Hadjiiski L, et al. Digital breast tomosynthesis: studies of the effects of acquisition geometry on contrast-to-noise ratio and observer preference of low-contrast objects in breast phantom images. Physics in Medicine & Biology. 2014;59(19):5883. doi: 10.1088/0031-9155/59/19/5883 25211509 PMC4264665

[45] Chen H, Zhang Y, Kalra MK, Lin F, Chen Y, Liao P, et al. Low-dose CT with a residual encoder-decoder convolutional neural network. IEEE transactions on medical imaging. 2017;36(12):2524–35. doi: 10.1109/TMI.2017.2715284 28622671 PMC5727581

[46] Su S, Delbracio M, Wang J, Sapiro G, Heidrich W, Wang O. Deep video deblurring for hand-held cameras. Proceedings of the IEEE Conference on Computer Vision and Pattern Recognition; 2017.

[47] Pathak D, Krahenbuhl P, Donahue J, Darrell T, Efros AA. Context encoders: Feature learning by inpainting. Proceedings of the IEEE conference on computer vision and pattern recognition; 2016.

[48] Yang C, Lu X, Lin Z, Shechtman E, Wang O, Li H. High-resolution image inpainting using multi-scale neural patch synthesis. Proceedings of the IEEE conference on computer vision and pattern recognition; 2017.

[49] Iizuka S, Simo-Serra E, Ishikawa H. Globally and locally consistent image completion. ACM Transactions on Graphics (ToG). 2017;36(4):1–14. doi: 10.1145/2897824.2925974

[50] Li Y, Liu S, Yang J, Yang M-H. Generative face completion. Proceedings of the IEEE conference on computer vision and pattern recognition; 2017.

[51] Tirada N, Li G, Dreizin D, Robinson L, Khorjekar G, Dromi S, Ernst T. Digital breast tomosynthesis: physics, artifacts, and quality control considerations. Radiographics. 2019;32(2):413–26. doi: 10.1148/rg.2019180046 30768362

[52] Mermuys K, Vanslambrouck K, Goubau J, Steyaert L, Casselman JW. Use of digital tomosynthesis: case report of a suspected scaphoid fracture and technique. Skeletal Radiology. 2008;37(6):569–72. doi: 10.1007/s00256-008-0470-3 18343919

[53] Pan SJ, Yang Q. A survey on transfer learning. IEEE Transactions on knowledge and data engineering. 2009;22(10):1345–59. doi: 10.1109/TKDE.2009.191

