# Supplementary material for: Two-phase learning-based 3D deblurring method for digital breast tomosynthesis images
Source: PLoS One. 2022 Jan 24;17(1):e0262736. doi: 10.1371/journal.pone.0262736 (PMC8786177; doi:10.1371/journal.pone.0262736)
Supplement: S1 File — (PDF) [file pone.0262736.s001.pdf]

Two-phase learning-based 3D deblurring method for digital breast tomosynthesis images: Supplementary Material

Yunsu Choi[1], Minah Han[1], Hanjoo Jang[1], Hyunjung Shim[1*], Jongduk Baek[1*]

1 School of Integrated Technology and Yonsei Institute of Convergence Technology, Yonsei University, 85, Songdo-gwahak-ro, Yeonsu-gu, Incheon, 21983, South Korea

* kateshim@yonsei.ac.kr * jongdukbaek@yonsei.ac.kr

Slice Thickness Filter

In this work, we did not use a slice thickness (ST) filter to keep the high-frequency components of the breast volume. When we used the ST filter, the high-frequency components were reduced, as shown in Figure 1(b), which is typical for breast tomosynthesis imaging. The ST filter is expressed as follows:

$$ST(f_z) = \begin{cases} 0.5[1+\cos(\frac{\pi f_z}{\sigma f_N})], & for\ |f_z| \prec \sigma f_N\ and |f_z| \prec \tan(\theta) f_N \\ 0, & elsewhere, \end{cases} \quad (1)$$

where, f_N is the Nyquist frequency, θ is the data acquisition angle, and σ was set to 0.4 as a multiplicative factor.

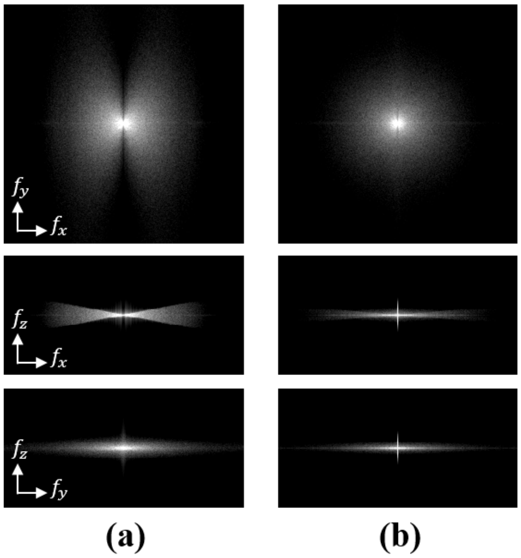

Fig 1. Frequency domain analysis. Frequency responses of DBT images using FDK (a) without and (b) with the ST filter for $f_x - f_y$ plane (Top), $f_x - f_z$ plane (middle), and $f_y - f_z$ plane (bottom). The display window is [1 4].

Phase 1 CNN

Batch normalization

We did not use the normalization layer because the batch normalization layer may degrade convolutional neural network (CNN) performance when the mini-batch size is small [1, 2]. We conducted network training with and without the normalization layer, and the results are presented in Figure 2. Our network is trained more stably when the batch normalization was excluded.

Filter number

We selected the filter number experimentally to achieve the best performance without sacrificing training efficiency. As depicted in Figure 3, validation loss converges when the number of filters is greater than 40. The validation loss converged to 0.0046 after 30 epochs with 40 and 48 filters. We also found that a network with 64 filters requires more training datasets for convergence.

Phase 2 CNN

We adopted the U-Net in phase 2, because it contains a large receptive field size to cover the length of PSF in the DBT system, which is a key aspect of the proposed method. When we used REDCNN or ResNet in phase 2, the performance of the deblurring was not effective compared to the case of U-Net as shown in Figure 4. Quantitative results summarized in Tables 1 and 2 also confirm these observations.

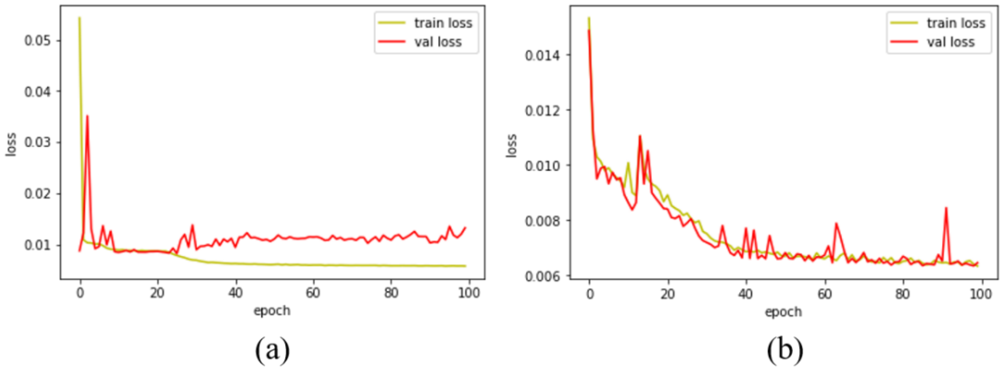

Fig 2. Training and validation loss in Phase 1. Training and validation loss in Phase 1 (a) with and (b) without the batch normalization layer.

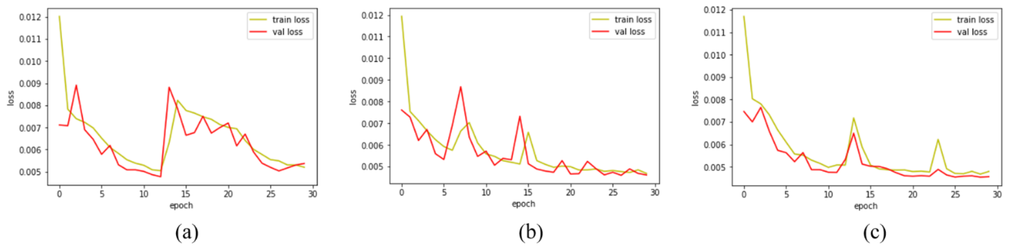

Fig 3. Filter numbers in Phase 1. Training and validation loss in Phase 1 when (a) 32 filters, (b) 40 filters, and (c) 48 filters are used.

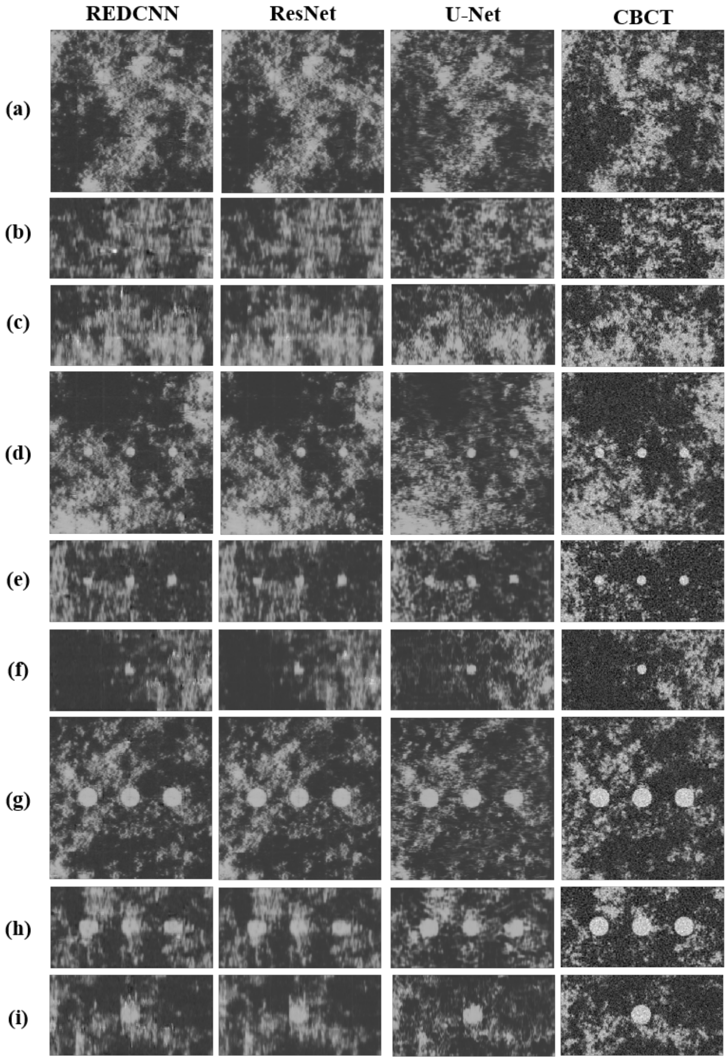

Fig 4. Results for various Phase-2 CNN. Deblurred images by the proposed method with REDCNN, ResNet, U-Net in Phase 2, and CBCT images (from left to right). Images without mass lesion for (a) axial, (b) coronal, and (c) sagittal planes; images containing 4 *mm* lesions for (d) axial, (e) coronal, and (f) sagittal planes, and images containing 2 *mm* lesions for (g) axial, (h) coronal, and (i) sagittal planes. The display window is [0.03 0.10] in cm^{-1}.

Table 1. MSE and GRMSE results for various Phase-2 CNN. (mean±standard deviation)

	MSE ($\times 10^{-5}$)			GRMSE ($\times 10^{-2}$)		
Method	Axial	Coronal	Sagittal	Axial	Coronal	Sagittal
U-Net	**7.09 ± 0.78**	**8.28 ± 1.32**	**7.56 ± 1.20**	**0.59 ± 0.01**	**0.59 ± 0.01**	**0.64 ± 0.01**
REDCNN	7.50 ± 0.61	10.05 ± 1.48	9.01 ± 1.22	0.69 ± 0.01	0.69 ± 0.01	0.77 ± 0.02
ResNet	7.50 ± 0.60	9.97 ± 1.50	8.87 ± 1.21	0.70 ± 0.01	0.70 ± 0.01	0.79 ± 0.02

Table 2. CNR results for various Phase-2 CNN. (mean±standard deviation)

	Contrast-to-Noise Ratio		
Method	Axial	Coronal	Sagittal
U-Net	**2.91 ± 0.79**	**2.42 ± 0.76**	**2.67 ± 0.73**
REDCNN	2.78 ± 0.77	**2.42 ± 0.70**	2.59 ± 0.88
ResNet	2.83 ± 0.83	2.38 ± 0.67	2.62 ± 0.88

Generalizability

To examine the generalization performance of the proposed algorithm, we generated 30% volumetric glandular fraction (VGF) DBT volume acquired over the ranges of $-40°$ to $40°$ and $-10°$ to $10°$ for the same breast volume. These two volumes were deblurred by the CNN pretrained with the DBT volume acquired over the range of $-20°$ to $20°$ and the corresponding cone-beam computed tomography (CBCT) volume pair. Figure 5 compares the deblurred images of the DBT volume for different data acquisition angles. The generalization performance is much better for the larger data acquisition angle (i.e., $-40°$ to $40°$). Because the primary role of the proposed method is to fill in the missing data for the DBT volume in the frequency domain (or equivalently, deblurring in image space), the generalization performance of the CNN for the DBT volumes acquired over $-10°$ to $10°$ data acquisition angles is worse because it contains much more missing data in frequency space. The quantitative results in Tables 3 and 4 also confirm these observations.

Further clarification

To the best of our knowledge, there have been no studies on 3D deblurring of DBT images using CNN yet. Instead, the PSF deblurring method based on iterative blind deconvolution (i.e., PSF deblur) [3] was compared with the proposed method (i.e.,

Table 3. MSE and GRMSE results of the DBT images with different data acquisition angles. (mean±standard deviation)

	MSE ($\times 10^{-5}$)			GRMSE ($\times 10^{-2}$)		
Method	Axial	Coronal	Sagittal	Axial	Coronal	Sagittal
$-40° \sim 40°$ PLMAE	**4.86 ± 0.66**	**6.66 ± 0.97**	**5.38 ± 1.10**	**0.56 ± 0.01**	**0.52 ± 0.02**	**0.59 ± 0.02**
$-10° \sim 10°$ PLMAE	12.77 ± 1.47	16.21 ± 2.35	14.16 ± 2.14	0.63 ± 0.01	0.60 ± 0.02	0.67 ± 0.02
$-40° \sim 40°$ FDK	16.54 ± 2.49			0.68 ± 0.03		
$-40° \sim 40°$ FDK	78.40 ± 13.16			1.63 ± 0.14		

Table 4. CNR results of the DBT images with different data acquisition angles. (mean±standard deviation)

	Contrast-to-Noise Ratio		
Method	Axial	Coronal	Sagittal
$-40° \sim 40°$ PLMAE	**2.87 ± 1.04**	**3.06 ± 0.99**	**3.39 ± 1.40**
$-10° \sim 10°$ PLMAE	1.76 ± 0.59	1.46 ± 0.75	1.37 ± 0.78
$-40° \sim 40°$ FDK	1.77 ± 0.42		
$-10° \sim 10°$ FDK	0.81 ± 0.36		

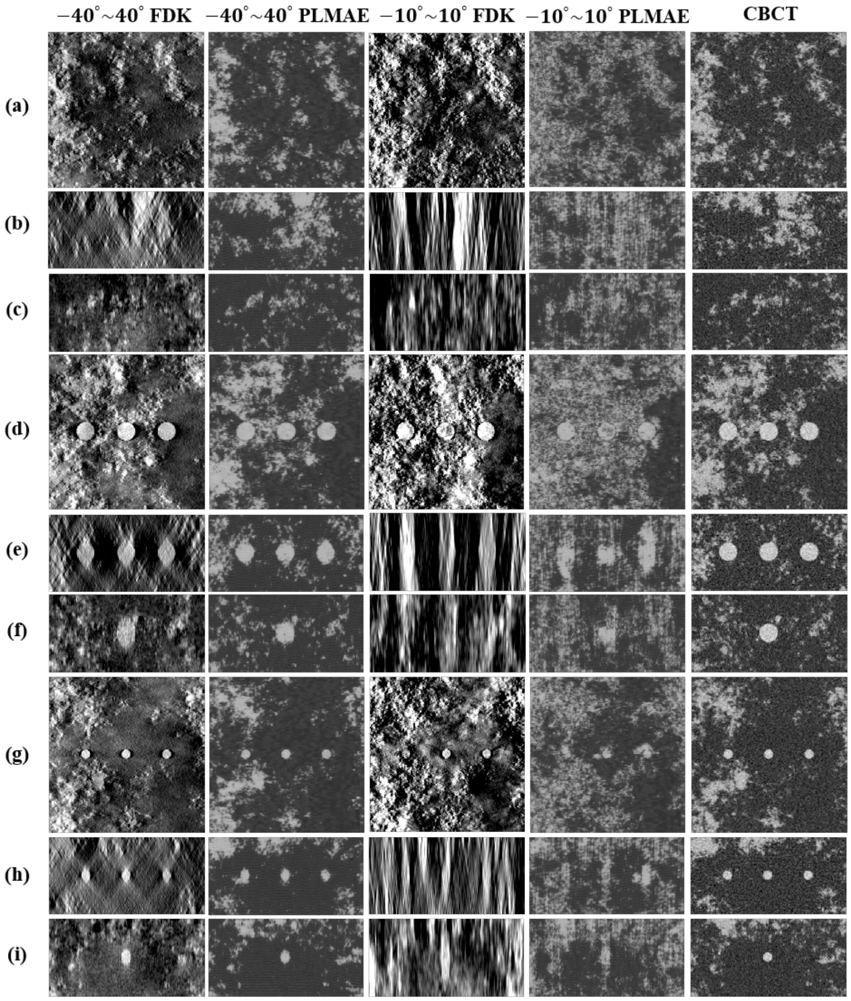

Fig 5. Results with different data acquisiton angle. Comparison of DBT images reconstructed by FDK over $-40°$ to $40°$ and $-10°$ to $10°$ data acquisition angles with deblurred images by the proposed method. CBCT images are shown as a reference. Images without mass lesion for (a) axial, (b) coronal, and (c) sagittal planes; images containing 4 mm lesions for (d) axial, (e) coronal, and (f) sagittal planes, and images containing 2 mm lesions for (g) axial, (h) coronal, and (i) sagittal planes. The display window is [0.03 0.10] in cm^{-1}.

PL-MAE). Among the several methods proposed in the PSF deblurring method [3], method 3, which had significantly improved SSIM with the reference image, was quantitatively compared with the proposed method. As illustrated in Figure 6, the image reconstructed by the PSF deblur method was not significantly different from the image reconstructed by the FDK. As shown in Table 6, the image deblured by the PSF deblur method slightly increased the CNR of 4 mm lesions compared to the image reconstructed by FDK, similar to the result of the previous study [3]. However, as shown in Table 5, the MSE and GRMSE between the PSF deblurred image and the reference image were increased compared to the image reconstructed by the FDK due to the increased noise level. These results demonstrate that our proposed method showed a higher performance than the PSF deblur method.

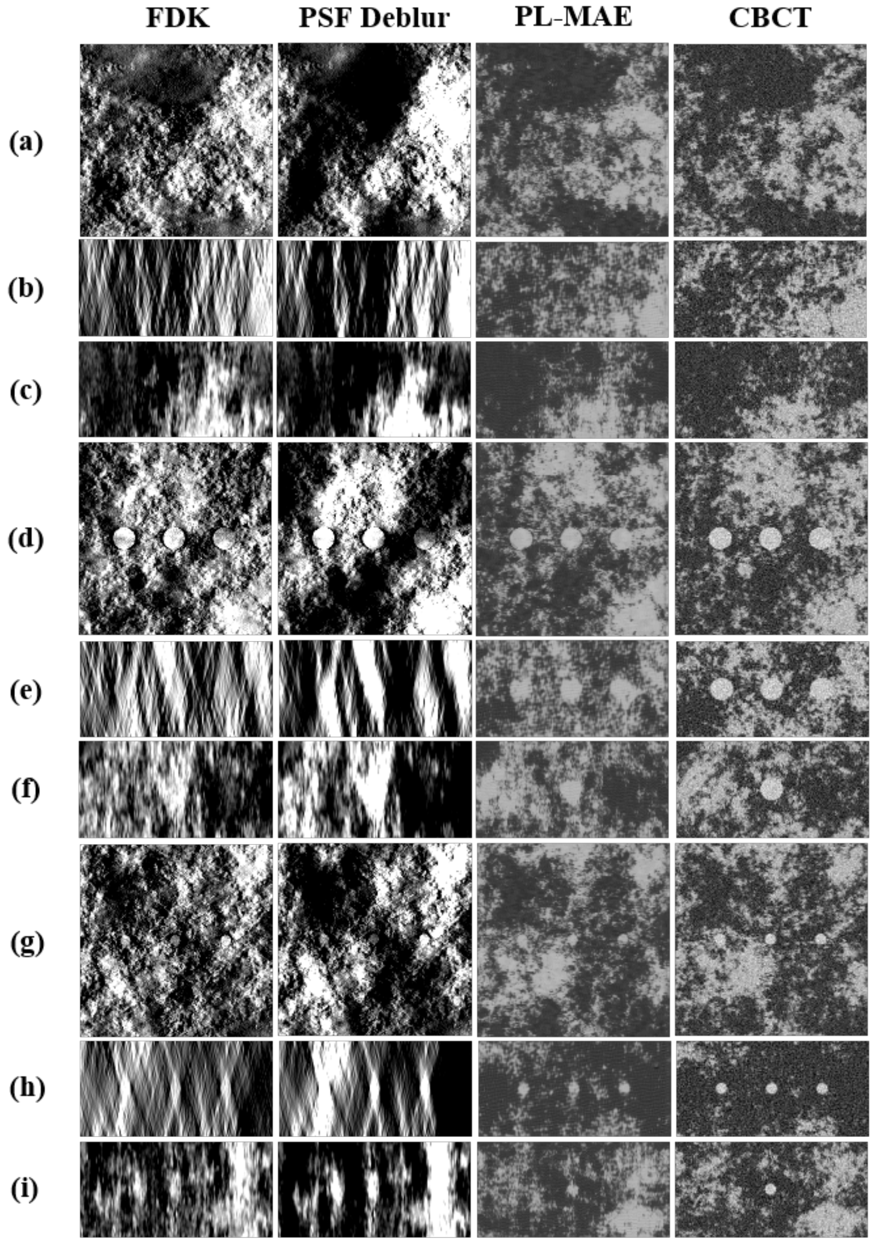

Fig 6. Results for further clarification. Comparison of DBT images reconstructed by FDK, deblurred images by PSF deblur method, deblurred images by the proposed method with PL-MAE loss function, and CBCT images (from left to right). Images without mass lesion for (a) axial, (b) coronal, and (c) sagittal planes; images containing 4 mm lesions for (d) axial, (e) coronal, and (f) sagittal planes, and images containing 2 mm lesions for (g) axial, (h) coronal, and (i) sagittal planes. The display window is [0.03 0.10] in cm^{-1}.

Table 5. MSE and GRMSE results of the DBT images reconstructed with FDK, deblurred images by PSF deblur method, and deblurred images by the proposed method with PL-MAE. (mean±standard deviation)

	MSE ($\times 10^{-5}$)			GRMSE ($\times 10^{-2}$)		
Method	Axial	Coronal	Sagittal	Axial	Coronal	Sagittal
PSF deblur	43.33 ± 13.3	55.87 ± 18.64	55.13 ± 17.56	1.11 ± 0.07	0.94 ± 0.07	0.80 ± 0.08
PL-MAE	**8.90 ± 1.30**	**10.02 ± 2.15**	**9.62 ± 2.06**	**0.59 ± 0.01**	**0.55 ± 0.01**	**0.64 ± 0.02**
FDK	41.09 ± 5.56			1.07 ± 0.06		

Table 6. CNR results of the DBT images reconstructed with FDK, deblurred images by PSF deblur method, and deblurred images by the proposed method with PL-MAE. (mean±standard deviation)

	Contrast-to-Noise Ratio		
Method	Axial	Coronal	Sagittal
PSF deblur	1.31 ± 0.69	1.63 ± 0.65	1.75 ± 0.67
PL-MAE	**3.24 ± 0.75**	**2.98 ± 1.05**	**3.19 ± 1.00**
FDK	1.14 ± 0.57		

Clinical Data

To verify that the proposed algorithm is effective for real imaging data, we used clinical chest data provided by the NIH clinical center. Table 7 summarizes the details of the simulation parameters. We observed that the proposed method performs effective 3D deblurring, significantly reducing the blurring artifacts in the in-focus plane and the other planes of the real imaging data. Quantitative results summarized in Table 8 also confirm these observations.

Table 7. Clinical Data Simulation Parameters.

Parameters	CBCT	DBT
Source to iso-center distance	800 mm	
Detector to iso-center distance	400 mm	
Data acquisition angle	$-180° \sim 180°$	$-45° \sim 45°$
Number of views	360	91
Detector cell size	$0.776 \times 0.776\, mm^2$	
Detector array size	768×512	
Reconstructed volume size	$264.9 \times 264.9 \times 264.9\, mm^3$	
Reconstructed voxel size	$0.517 \times 0.517 \times 0.517\, mm^3$	
Reconstruction algorithm	FDK	

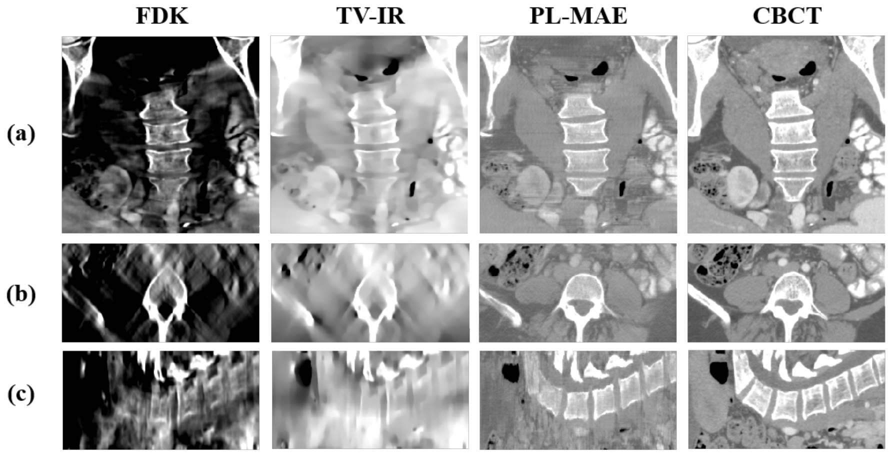

Fig 7. Results with clinical data. NIH chest images reconstructed with FDK and TV-IR, deblurred images by the proposed method with PL-MAE loss function, and CBCT images (from left to right) for (a) axial, (b) coronal, and (c) sagittal planes. The display window is [-450 450] in Hounsfield unit (HU).

Table 8. MSE and GRMSE results of the clinical DT images reconstructed with FDK, deblurred images by PSF deblur method, and deblurred images by the proposed method with PL-MAE. (mean±standard deviation)

	MSE ($\times 10^4$)			GRMSE		
Method	Axial	Coronal	Sagittal	Axial	Coronal	Sagittal
PL-MAE	**0.56 ± 0.23**	**0.87 ± 0.35**	**0.92 ± 0.25**	**27.83 ± 5.67**	**37.84 ± 8.14**	**43.14 ± 7.48**
FDK	13.13 ± 6.74			31.64 ± 7.53		
TV-IR	1.88 ± 0.96			36.04 ± 8.27		

References

1. Ioffe S. Batch renormalization: Towards reducing minibatch dependence in batch-normalized models.. arXiv preprint arXiv:170203275. 2017.

2. Singh S, Shrivastava A. Evalnorm: Estimating batch normalization statistics for evaluation.. Proceedings of the IEEE/CVF International Conference on Computer Vision; 2019.

3. Mota AM, Clarkson MJ, Almeida P, Matela N. An Enhanced Visualization of DBT Imaging Using Blind Deconvolution and Total Variation Minimization Regularization.. IEEE Trans Med Imaging. 2020;39(12):4094-101.
